# Shear performance evaluation of sustainable concrete beams containing rice husk ash and carbon nanotubes as cement replacement

**DOI:** 10.1371/journal.pone.0320325

**Published:** 2025-06-04

**Authors:** Yi Jing, Jin Chai Lee, Wei Chek Moon, Jing Lin Ng, Ming Kun Yew, Yong Jin

**Affiliations:** 1 Department of Civil Engineering, Faculty of Engineering, Technology and Built Environment, UCSI University, Kuala Lumpur, Malaysia; 2 School of Civil Engineering, College of Engineering, Universiti Teknologi MARA, Shah Alam, Malaysia; 3 Lee Kong Chian Faculty of Engineering and Science, Universiti Tunku Abdul Rahman, Cheras, Kajang, Malaysia; 4 Department of Civil Engineering, School of Architecture and Energy Engineering, Wenzhou University of Technology, Wenzhou, Zhejiang, China; SASTRA Deemed University, INDIA

## Abstract

The utilization of rice husk ash (RHA) as a cement substitute in concrete can mitigate the environmental issues caused by concrete production. This study is the first investigated the effect of different concentrations of multiwall carbon nanotubes (MWCNTs) on the shear behaviour of RHA-reinforced concrete (RC) beams. Three RHA RC beams incorporating different MWCNTs concentrations (0%, 0.1%, and 0.2%) were subjected to shear failure tests, with shear behaviour evaluated based on load-deflection capacity, failure load, strain distribution, and crack development morphology. The results indicate that the incorporation of well-dispersed MWCNTs (0.1%) significantly enhances the shear resistance of RHA RC beams. Specifically, with the addition of 0.1% MWCNTs, the first crack load, initial stiffness, and ultimate shear capacity increased by 34.78%, 38.4%, and 4.76%, respectively, while midspan deflection and ductility improved by 23.19% compared to the control specimen. However, excessive MWCNTs incorporation negatively impacted shear capacity, as the inclusion of 0.2% MWCNTs led to an 8.57% reduction in the ultimate shear capacity of the RHA RC beam. Additionally, the experimental results were compared with predictions from several models, including the ACI 318 model, Eurocode 2–05 model, Canadian standard, Bazant and Kim, Zsutty, and Collins and Kuchma models. The comparison revealed that the Eurocode 2–05 model, Canadian standard, and Zsutty models provide reliable predictions of the shear behaviour of RHA RC beams, with an error margin within 20%. The findings suggested that well-dispersed MWCNTs can play a role in improving the initial stiffness and ductility of RHA RC beams, enhancing the shear resistance of the beams, delaying shear failure, and preventing brittle failure of the beams. Furthermore, the improvement of the performance of RHA sustainable concrete components by MWCNTs contributes to the sustainable development of the construction industry.

## 1. Introduction

### 1.1. Background

Concrete components are widely utilised in the construction industry due to their excellent mechanical properties and durability. However, concrete production poses significant environmental challenges. The primary source of aggregates in concrete is non-renewable natural stone, while cement, its key constituent, is produced in vast quantities. Since the 2010s, the rapid expansion of the global construction industry has driven a substantial increase in cement production, with annual output averaging over 4 billion tonnes in the past decade [1]. Cement manufacturing is a major contributor to carbon dioxide emissions, accounting for approximately 7% of global emissions, with severe consequences for the global ecosystem [2]. To address the environmental impact of concrete production, researchers have explored various solutions, including geopolymer concrete, lightweight concrete, and sustainable concrete [3–6]. A fundamental approach to reducing these environmental issues is the development of viable substitutes for cement and aggregates. The utilisation of supplementary cementitious materials (SCMs) has significantly reduced greenhouse gas (GHG) emissions from cement production [7]. Common SCMs are classified as either industrial by-products (e.g., fly ash, blast-furnace slag, silica fume, and metakaolin) or agricultural by-products (e.g., rice husk ash (RHA), olive waste ash, corncob ash, and bagasse ash) [8–15]. Similarly, alternative aggregate materials have been explored, including perlite, lightweight expanded clay aggregate, waste nylon, coal waste, and waste polyethylene terephthalate [16–20]. Among these, RHA is one of the most readily available agricultural waste materials in Asia, offering distinct regional advantages for use in the construction industry. Consequently, it has attracted considerable attention from researchers.

Asia is the world’s leading region for rice production and consumption, accounting for approximately 90% of global rice output. China is the largest producer, yielding 206.6 million tonnes of rice in 2022, followed by India and Indonesia, which produced 196.25 and 54.75 million tonnes, respectively [21,22]. RHA, a byproduct of the high-temperature incineration of rice husks, poses significant environmental risks when disposed of indiscriminately, as it can harm ecosystems, pollute agricultural land, and pose health hazards [23]. However, when processed under optimal conditions—specifically, controlled combustion temperatures between 500°C and 700°C—RHA becomes an effective supplementary cementitious material due to its high amorphous silica content (over 80%) [24,25]. The amorphous silica in RHA exhibits pozzolanic activity, reacting with Ca(OH)₂ released during cement hydration to form hydrated calcium silicate (C-S-H) gel. This reaction enhances the internal structure of cement-based materials, improving their overall performance [26,27]. Extensive research has been conducted on the mechanical and durability properties of concrete containing RHA. Balapour et al. (2017) [28] examined the effects of incorporating up to 15% nano- and micro-RHA on the compressive strength and electrical resistivity of mortars. Their findings revealed that, compared to control specimens without RHA, the 90-d compressive strength increased by 36.18%, while the 28-d electrical resistivity improved by 147.45%. Similarly, Faried et al. [29] investigated the influence of RHA produced at varying combustion temperatures (300°C to 900°C) and durations (3–9 h) on the mechanical and durability properties of concrete. The results indicated that RHA produced at 700°C for 5 h yielded the most significant performance improvements. Compared with the control group, the addition of 3% RHA increased compressive strength by 21.9% and splitting tensile strength by 26.67%, while sorptivity decreased from 5*10^-6^ mm/sec^0.5^ to 0.33*10^-6^ mm/sec^0.5^. Beyond investigations into the mechanical properties, durability, and functionality of RHA-incorporated concrete, some researchers have focused on the performance of reinforced concrete (RC) beams containing RHA. Osman et al. [30] studied the shear behavior of ultra-high performance fibre-reinforced concrete (UHPFRC) beams using RHA, sugar cane bagasse ash, and fly ash as SCMs. Their results showed that a 10% RHA replacement level optimised the shear behavior of UHPFRC beams, leading to comprehensive improvements in shear strength, stiffness, and toughness. Among them, the shear strength was improved by 15%. Hassanean et al. (2024) [31] investigated the shear behaviour of RC beams incorporating varying percentages of RHA (up to 20%) and found that replacing 10% of cement with RHA significantly enhanced the shear capacity, stiffness, and toughness of the RC beams.

To expand the application of RHA-based sustainable concrete in the construction industry and further enhance its overall performance, researchers have introduced nanoparticles into RHA concrete. Common nanoparticles used in RHA concrete include nano-SiO_2_, nano-Al_2_O_3_, and nano-TiO_2_ [32–34]. The performance enhancement of cement-based materials by nanoparticles is primarily attributed to their nucleation, bridging, filling, and pozzolanic activity [35–37]. Anto et al. [38] reported that the combined incorporation of 3% nano-SiO_2_ and 10% RHA resulted in significant improvements in compressive strength, flexural strength, water sorption resistance, and acid attack resistance. Similarly, Avudaiappan et al. [33] observed that the addition of 1.0% nano-SiO_2_ and 10% RHA increased the 28-d compressive strength, flexural strength, and splitting tensile strength from 24.5 MPa, 3.71 MPa, and 2.56 MPa to 31.3 MPa, 4.2 MPa, and 3.28 MPa, respectively. Additionally, the synergistic effect of RHA and well-dispersed nano-SiO_2_ effectively reduced the porosity of the concrete. Alex et al. [34] observed that incorporating 10% micro RHA and 1% nano-Al₂O₃ increased the 28-d compressive strength and splitting tensile strength of cement mortar by 26.8% and 48.72%, respectively, while also significantly reducing water absorption. Meddah et al. [39] reported that the nucleation, pozzolanic activity, and filler effect of nano-Al₂O₃ accelerated cement hydration, generated additional C-S-H gel, filled capillary pores, and compacted the internal structure of RHA-incorporated concrete, thereby enhancing its mechanical properties. Praveenkumar et al. [32] demonstrated that the addition of 3% nano-TiO_2_ and 10% RHA significantly improved the overall performance of concrete. Compared with the control specimen, the 28-d compressive strength increased by 11%, while water absorption, porosity, and chloride ion penetration were notably reduced. This improvement is attributed to the synergistic effect of RHA’s pozzolanic properties and nano-TiO₂’s filler effect, which effectively refines the pore structure and densifies the cement matrix [40].

The failure of concrete structural elements poses a significant risk to building safety [41,42]. To reduce the risks associated to concrete structural failure, researchers have suggested multiple approaches to improve the structural performance of concrete. The use of concrete-filled steel tubes can diminish the brittleness of concrete components and enhance the ductility of concrete constructions [43–45]. The utilization of fiber-reinforced polymer (FRP) bars can improve bond strength, thus enhancing the mechanical performance of concrete structural elements. Furthermore, the corrosion-resistant characteristics of FRP bars can augment the durability of concrete structural elements in harsh situations [46–49]. Crack damage is one of the main reasons for the reduction of concrete structure performance, and the incorporation of fiber materials into concrete has been proven to effectively control the development of cracks [50,51]. The fibers typically utilized in concrete comprise steel fibers, polyethylene fibers, glass fibers, carbon fibers, and carbon nanotubes, etc [52–56]. Among them, CNTs, cylindrical nanomaterials formed by carbon allotropes, can effectively control the development of microcracks due to their excellent physical properties and high aspect ratio [57–60]. Demczyk et al. [61] reported that the tensile strength and elastic modulus of multiwall carbon nanotubes (MWCNTs) can reach up to 0.15 TPa and 0.9 TPa, respectively. The strengthening effect of CNTs in cement-based materials is primarily attributed to their nucleation, filling, and bridging mechanisms, which promote the formation of C-S-H gel, effectively densify the internal pore structure, and inhibit the development of microcracks [62,63]. Sarvandani et al. [64] studied the effect of COOH-MWCNTs on the mechanical and durability properties of cement mortar in sulfate solutions and saturated limewater environments. Their findings showed that incorporating 0.2% COOH-MWCNTs increased the 180-d compressive strength of cement mortar in a saturated limewater environment by 22.1%. Additionally, in a sulfate environment, the 360-d compressive strength loss and flexural strength loss decreased by 13.37% and 14.08%, respectively, while the 90-d capillary water absorption rate was reduced by 39.2%. Yao and Lu [65] investigated the mechanical properties of concrete incorporating various CNT concentrations at different temperatures (room temperature, 300°C, 600°C, and 900°C). Their results indicated that with 0.5 wt% CNTs, the 28-d compressive strength at room temperature and the residual compressive strength at 300°C reached peak values compared to control specimens without CNTs. Recent research has also explored the application of CNTs in RC beams. Irshidat [66] observed that incorporating 0.2% CNTs enhanced the compressive and splitting tensile strengths of concrete by 23% and 22%, respectively. Furthermore, CNTs improved the bond strength and stiffness between steel reinforcement and concrete. Hassan et al. [67] reported that well-dispersed CNTs significantly enhanced the bond strength of 12 mm and 16 mm diameter steel bars, with increases of 36% and 21%, respectively. Öztürk. [68] studied the engineering properties of geopolymer concrete beams containing a small amount of CNTs or carbon black (CB) under mixed failure modes (shear and flexural). The results showed that compared with the CB-based geopolymer beam, the loading-carrying capacity, initial stiffness, and energy dissipation capacity of the CNT-based geopolymer beam increased from 15.2 KN, 2.6 KN/mm, and 113.14 KN.mm to 27.8KN, 3.2 KN/mm, and 369.14 KN.mm, respectively.

In summary, the production of sustainable concrete should not only focus on minimising its environmental impact but also on enhancing its overall performance compared to conventional concrete, thereby broadening its potential applications. In the case of RHA-based sustainable concrete, researchers have explored the incorporation of various nanoparticles to develop high-performance RHA concrete. However, despite the exceptional microcrack control capabilities of CNTs, their application in RHA concrete remains largely unexplored. Notably, no studies have yet investigated the use of CNTs in RHA concrete structural components.

Given these considerations, this study is the first to evaluate the effect of different MWCNTs concentrations on the shear performance of RHA-modified concrete beams without shear reinforcement. By systematically varying the MWCNTs content (0% to 2%), a series of modified RHA RC beams were prepared, and their shear behaviour was analysed in terms of crack propagation, failure modes, shear capacity, load-deflection curves, and load-strain responses.

### 1.2. Research significance and novelty

To reduce the environmental impact of concrete production, RHA has been widely recognised as a low-carbon alternative to cement. While numerous studies have explored ways to enhance the overall performance of RHA concrete, the majority have focused on its mechanical properties and durability, with relatively little research on its application in structural components. At the same time, MWCNTs, due to their excellent material properties, have promoted the mechanical and durability characteristics of cement-based materials, particularly in terms of crack control [69]. To expand the application of RHA sustainable concrete, particularly in structural elements, research on MWCNTs-modified RHA concrete components is essential. This study addresses this gap by evaluating the shear behaviour of RHA RC beams containing MWCNTs, providing a foundation for the potential use of RHA concrete in structural applications and contributing to the reduction of environmental issues in the construction industry.

## 2. Materials and methods

### 2.1. Materials

Ordinary Portland cement (OPC) Type 42.5, with a specific surface area of 3430 cm^2^/g and a specific gravity of 3.14, was used as the binder. Crushed limestone with particle sizes ranging from 5 to 20 mm served as the coarse aggregate, while river sand with a particle size range of 0–4.75 mm and a fineness modulus of 2.9 was used as the fine aggregate. A polycarboxylate ether (PCE) superplasticiser, offering a water reduction rate of 28%, was employed. Rice husks sourced from Xuzhou, China, were incinerated in a furnace at 800°C for 60 min to produce RHA, which consisted of 87.3% silicon dioxide (SiO_2_), 2.8% potassium sulphate (K_2_SO_4_), and 1.3% calcium carbonate (CaCO_3_). The physical properties of MWCNTs are presented in Table 1.

**Table 1:** Physical properties of MWCNTs.

Purity	>97.5%
Diameter	7–15 nm
Length	5–15 μm
Specific surface area	286.3 m^2^/g
Density	0.038 g/cm^3^

### 2.2. Mix proportions and specimen preparation

Table 2 presents the mixture proportions. Aggregates, cement, and RHA were sequentially added to the mixer and blended for 2 min. Subsequently, the PCE, uniformly dispersed MWCNTs, and the remaining water were introduced and mixed for an additional 5 min. To ensure effective dispersion, an ultrasonic method was employed. The MWCNTs aqueous solution, contained in a beaker, was ultrasonicated for 30 min using a 750 W ultrasonic disperser with a cycle of 3 s on and 3 s off. To maintain the solution at room temperature, the beaker was submerged in a cold-water container, which was replenished with fresh water every 10 min.

**Table 2:** Mixture proportions.

Mix Code	Beam	Binder			Coarse Aggregate	Fine Aggregate	Water	Additive
Cement	RHA	CNT
R15C0	B1	448.97	79.23	0	1028.7	712.85	169	2.5%
R15C10	B2	448.44	79.23	0.53	1028.7	712.85	169	2.5%
R15C20	B3	447.92	79.23	1.05	1028.7	712.85	169	2.5%

**Fig 1:**
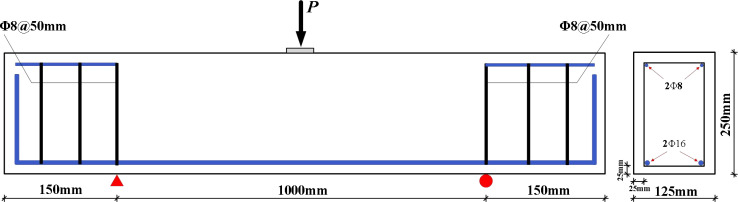
Reinforcement arrangement of beams.

Three RC beams, each measuring 1000 mm × 125 mm × 250 mm and prepared according to the mix proportions specified in Table 2, were used in this study. All steel reinforcements were obtained from a single supplier. HRB500 bars (hot-rolled ribbed steel bars) with a 16 mm diameter were used for flexural reinforcement. Shear reinforcement was provided at the beam supports over a 150 mm span (at 50 mm intervals) using HPB300 bars (hot-rolled plain steel bars) with an 8 mm diameter. The mechanical properties of these steel bars are presented in Table 3. Additionally, 100 mm cube specimens were prepared to assess the 28-d compressive strength following the GB/T 50080–2019 test method [70]. The properties of all beams are summarised in Table 4. Both the beams and specimens were cured under standard conditions. The reinforcement arrangement of the beams is shown in Fig 1.

**Table 3:** Mechanical properties of steel bars.

Steel bar	Yield strength (MPa)	Ultimate strength (MPa)	Elongation (%)
HRB500 bars	547.1	720.8	15.2%
HPB300 bars	468.2	658.4	16.9%

**Table 4:** Properties of beam.

Beam	Clear cover (mm)	Constant tensile reinforcement ratio	Shear span (mm)	Effective depth (mm)	Shear span/effective depth ratio
B1	25	1.52%	150	209	2.39
B2					
B3					

### 2.3. Test method

**Fig 2:**
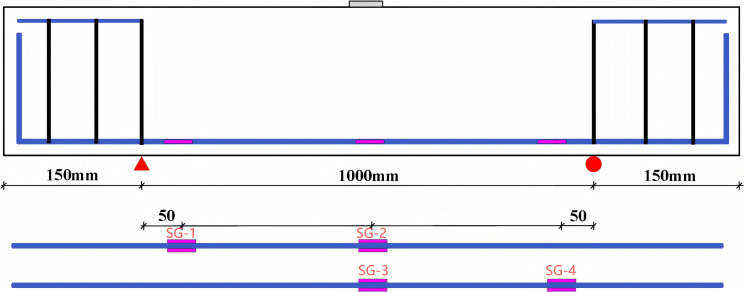
Locations of strain gauges on stressed steel bars.

**Fig 3:**
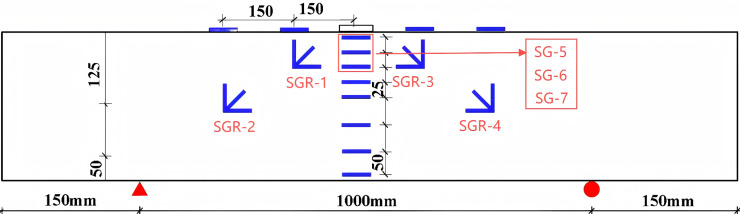
Locations of strain gauges on beam.

The shear behaviour of the beams was tested in accordance with GB/T 50152-2012 [71]. Data collection was automated using a static resistance strain gauge connected to a dynamic signal acquisition and analysis system. Testing was performed on a universal testing machine with a maximum capacity of 600 kN. Midspan deflection was measured using a linear voltage differential transducer (LVDT). A graded loading system with 12 load levels was employed, with the initial load applied at each interval using load control at a rate of 5 kN/min. To evaluate beam deformation under different loads, strain gauges were affixed to the stressed steel bars and beam surfaces during specimen preparation, while displacement meters were installed at designated locations on the specimens. Figs 2 and 3 illustrate the positions of the strain gauges on the stressed steel bars and beams, respectively. This study analysed the strains in the steel bars (SG-1 to SG-4) and in the compression zone of the midspan concrete (SG-5 to SG-7). Additionally, four groups of strain gauge rosettes (SGR1 to SGR4), each consisting of three strain gauges arranged at angles of 0°, 45°, and 90°, were positioned in a straight line from the loading point to the support to measure the principal strains in the shear span. Once the load reached 60 kN, loading was continued until specimen failure. Furthermore, shear crack widths were measured after each loading level using a crack-width meter or comparison card, with an accuracy of 0.02 mm.

## 3. Results and discussion

### 3.1. Crack behaviour

**Fig 4:**
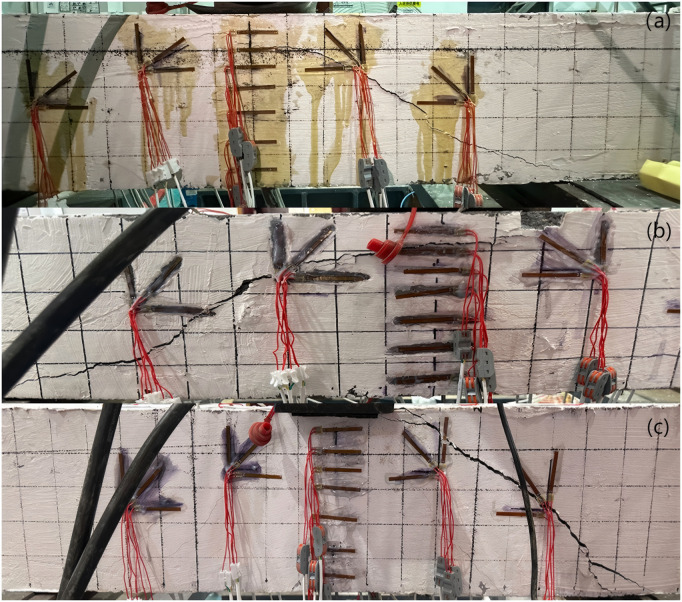
Crack pattern at failure of beams: (a) B1, (b) B2, (c) B3.

Fig 4 illustrates the crack patterns observed at the failure of all beams, while Table 5 presents the experimental results, which are discussed in the following section.

**Table 5:** Experimental results of all beams.

Beam	28-d compressive strength (MPa)	First crack load (KN)	Midspan Defection at first crack load (mm)	Initial stiffness (KN/mm)	Yielding load (KN)	Mid-span Defection at yielding load (mm)	Ultimate load (KN)	Mid-span Defection at Ultimate load (mm)	Ultimate shear capacity (KN)	Ductility
B1	70	46	3.38	12.11	78.75	5.13	105	6.5	52.5	1.38
B2	76.1	62	3.7	16.76	82.5	5.07	110	7	55	1.70
B3	57.3	46	3.18	14.38	72	5.1	96	7.1	48	1.92

For Beam B1, no significant phenomena were observed during the initial loading phase. The first crack appeared at a load of 46 kN at the bottom midspan, measuring 4 cm in length and 0.2 mm in width. As the load increased to 52 kN, this midspan crack extended upwards, and a new crack emerged at the left support, measuring 6 cm in length and 0.2 mm in width. This crack extended diagonally towards the loading point, progressively widening. At 58 kN, both the midspan crack and the crack at the left support continued to propagate upwards, while a new crack, 8 cm in length and 0.47 mm in width, appeared at the right support. When the load reached 68 kN, a branch crack formed on the left primary crack, measuring 5 cm in length and 0.65 mm in width. Upon reaching 80% of the ultimate load, the existing cracks continued to extend along their original paths, with minimal formation of new cracks. At the ultimate load of 105 kN, the maximum crack width reached 1.6 mm, extending from the support to the load point, ultimately causing failure and significantly reducing the beam’s load-bearing capacity.

For Beam B2, the first crack appeared at a load of 62 kN at the bottom left support, measuring 4 cm in length and 0.18 mm in width. At 68 kN, another crack formed at the bottom right support, measuring 6 cm in length and 0.25 mm in width. The load was applied in 5 kN increments. As loading progressed, the existing cracks widened, but no additional cracks developed. At the failure threshold of 110 kN, the maximum crack width reached 1.8 mm.

For Beam B3, the first crack appeared at a load of 46 kN at the bottom left support, with a length of 3 cm and a width of 0.25 mm. At 53 kN, an oblique crack developed at the bottom right support, measuring 7 cm in length and 0.32 mm in width. As the load increased, cracks on both sides of the support extended diagonally from the fracture point towards the top of the loading point, with their widths progressively widening. At the failure load of 96 kN, the maximum crack width reached 1.74 mm, marking the beam’s load-bearing limit.

**Fig 5:**
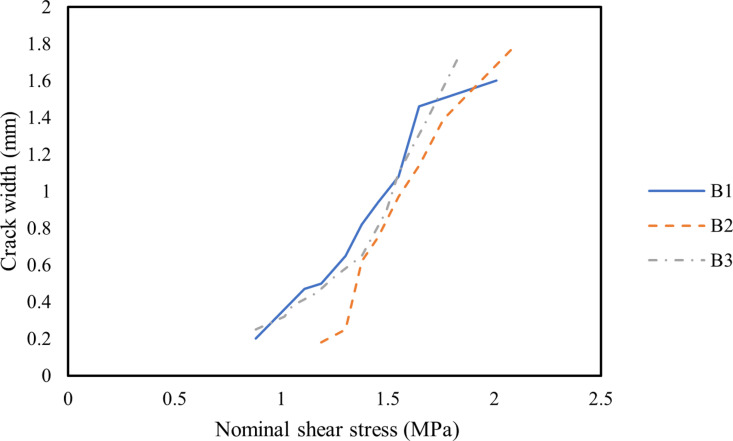
Relationship between main diagonal crack width and nominal shear stress of all beams.


Fig 5 illustrates the relationship between the main diagonal crack width and nominal shear stress, calculated using the following equation:

$$Nominal\;shear\;stress=\frac{V}{bh_{0}};V=F/2,$$

where$\;h_{0}$ denotes the effective depth (mm).

As shown in Fig 5, the main diagonal crack development rates were similar for all beams. However, under the same nominal shear stress, the inclusion of MWCNTs effectively reduced crack width during crack propagation. For example, at a nominal shear stress of 1.18 MPa, the maximum main diagonal crack widths for Beams B1, B2, and B3 were 0.5 mm, 0.18 mm, and 0.45 mm, respectively. This reduction is primarily attributed to the bridging effect of MWCNTs, which effectively delayed crack propagation [72]. Similar findings were reported by Naji et al. [73], who observed that the addition of MWCNTs increased the ultimate load and reduced the number of cracks due to their bridging effect, which hindered crack propagation.

### 3.2. Failure mode

The cracking patterns of all beams were generally similar, exhibiting pure shear failure, prominent diagonal fissures, and signs of concrete crushing in the upper section. Upon unloading, the test beams displayed considerable rebound. As shown in Table 5, the incorporation of MWCNTs enhanced the initial stiffness of the RHA RC beams, increasing it by 38.8% for Beam B2 and 13.7% for Beam B3 compared to Beam B1. Furthermore, the inclusion of 0.1% MWCNTs led to a 34.8% increase in the first crack load and a 4.8% improvement in the ultimate shear capacity of Beam B2 relative to Beam B1. The 28-d compressive strength of Beam B2 also increased by 8.7% compared to Beam B1, confirming that the incorporation of MWCNTs improved the mechanical properties of RHA concrete. The enhancement in 28-d compressive strength, initial stiffness, first crack load, and ultimate shear capacity with the addition of 0.1% MWCNTs can be attributed to the following mechanisms:

1. Nucleation effect − MWCNTs slow the early coverage of C-S-H gel on unhydrated cement and RHA particles, thereby enhancing the pozzolanic reaction between cement and RHA particles and densifying the internal structure of the concrete.
2. Filler effect − Due to their nanoscale characteristics, MWCNTs effectively fill the capillaries in cement-based materials, reducing the number of macropores and capillaries while promoting the formation of high-rigidity C-S-H gel.
3. Bridging effect − MWCNTs can effectively bridge microcracks and improve the interface transition zone (ITZ) [58,74–77].

Lee et al. [78] reached a similar conclusion in their study on the effect of CNTs on the shear behaviour of polyvinyl alcohol-engineered cementitious composite beams. Their findings indicated that the incorporation of well-dispersed CNTs increased shear capacity by up to 33% and enhanced the initial stiffness of the beams. However, when the MWCNTs concentration reached 0.2%, the 28-d compressive strength and ultimate shear capacity of Beam B3 significantly decreased by 18.14% and 8.57%, respectively, compared to Beam B1. This reduction is likely due to the re-agglomeration of MWCNTs at high concentrations. The clustering of MWCNTs around cement grains inhibits hydration reactions, increases porosity, and introduces internal defects in the cement matrix, ultimately reducing the mechanical properties of RHA concrete [69,79–81].

### 3.3. Load-deflection curves

**Fig 6:**
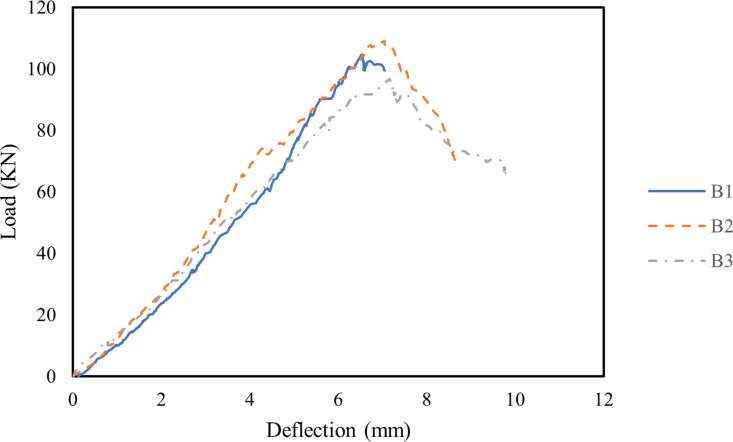
Load-deflection curves of all beams.

The load-deflection curves for the three beams are shown in Fig 6. As illustrated in Fig 6 and Table 5, the addition of MWCNTs significantly improved the ductility of the RHA RC beams. Specifically, with 0.1% MWCNTs, the ultimate load of Beam B2 increased to 110 kN, with a corresponding midspan deflection of 7.0 mm, whereas Beam B1 exhibited a midspan deflection of 6.5 mm at its ultimate load of 105 kN. This improvement can be primarily attributed to the bridging and filling effects of well-dispersed MWCNTs, which interact synergistically with the pozzolanic reaction of RHA. This interaction promotes the formation of high-stiffness C-S-H within the cement matrix, thereby enhancing the load-bearing capacity of the RHA RC beam [75,82]. Additionally, the incorporation of MWCNTs contributes to energy absorption by inhibiting microcrack development, thereby increasing the energy required for crack propagation and enhancing the ductility of RHA RC beams [73,83]. Similar results were reported by Qissab and Abbas [84], where the addition of 0.03–0.06% long and short MWCNTs enhanced the ductility of RC beams compared to beams without MWCNTs. Kanagaraj et al. [85] observed that the incorporation of MWNCTs enhanced the ductility of geopolymer concrete beams. The ductility index of geopolymer concrete beams rose from 1.42 to 1.93 with an addition of 0.16% MWNCTs relative to the control specimen. However, excessive MWCNTs content (0.2%) led to agglomeration, reducing the load-carrying capacity of the RHA RC beams. Although the midspan deflection of Beam B3 at its ultimate load was 7.1 mm, its ultimate load was 96 kN—approximately 87.3% of that of Beam B2.

### 3.4. Strain in longitudinal reinforcement

**Fig 7:**
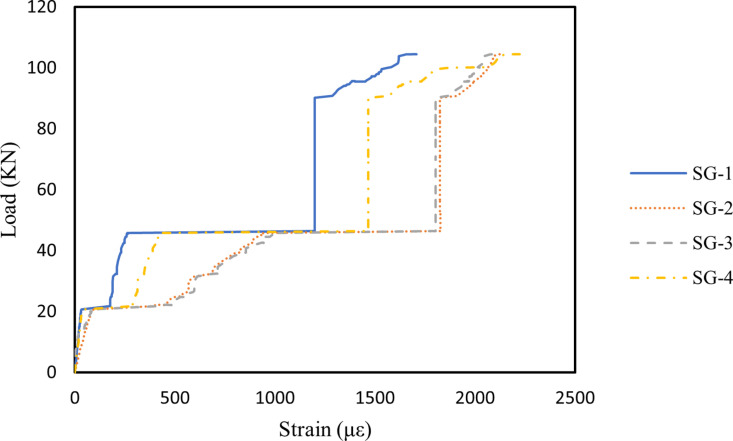
Steel bars strain of B1 beam.

**Fig 8:**
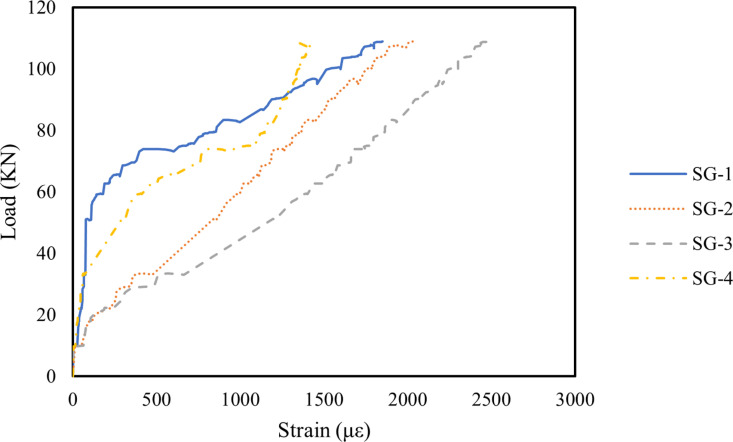
Steel bars strain of B2 beam.

**Fig 9:**
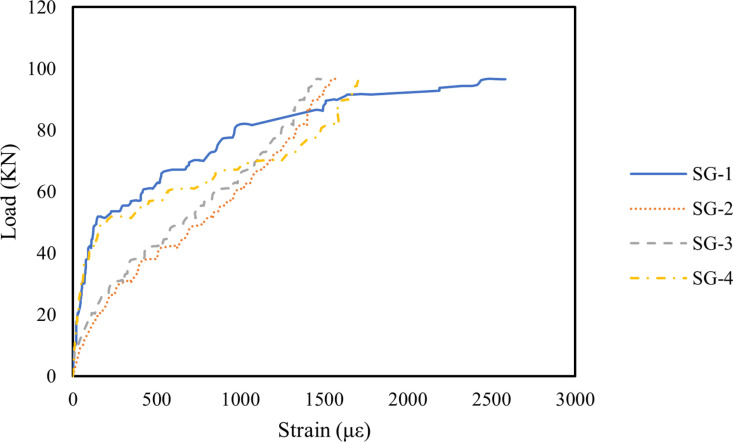
Steel bars strain of B3 beam.

Figs 7–9 illustrate the strain variations in the longitudinal reinforcements of Beams B1, B2, and B3 under applied loads. The strain patterns were similar across all three beams: as the load increased, the strain in the longitudinal steel bars at various locations also increased. Initially, strain development at each measurement point was minimal. However, with increasing load, the strain at SG-2 and SG-3 grew significantly, indicating higher tension in these steel bars due to the bending moment. The strain values at SG-1 and SG-4 rose sharply as oblique cracks formed near the supports, reflecting stress redistribution in the steel bars in the cracked regions once the concrete fractured [86]. In Fig 8, the incorporation of 0.1% MWCNTs in Beam B2 enabled the longitudinal steel bars to sustain a slower increase in strain under higher loads compared to Beam B1. This suggests that MWCNTs enhance the crack resistance of concrete, delaying crack formation [87]. A similar observation was reported by Irshidat and Al-Shannaq [88], who observed that CNT incorporation significantly increased the initial stiffness of RC beams. However, as shown in Fig 9, when the MWCNTs concentration increased to 0.2%, the strain in the steel bars rose rapidly under a relatively lower load than in Beam B2. This phenomenon is attributed to MWCNTs agglomeration at higher concentrations, which increases porosity in the RHA concrete, weakens the bond between the concrete and steel bars, and accelerates crack initiation [67,89–91].

### 3.5. Strain in concrete

#### 3.5.1. Strain on midspan of concrete.

**Fig 10:**
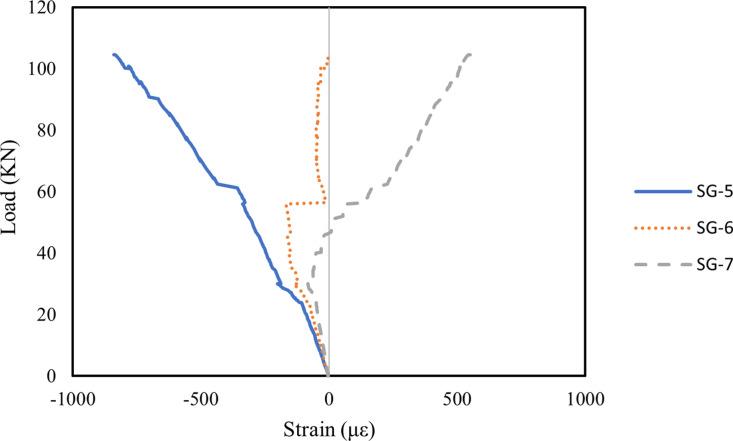
Strain on midspan of concrete in B1 beam.

**Fig 11:**
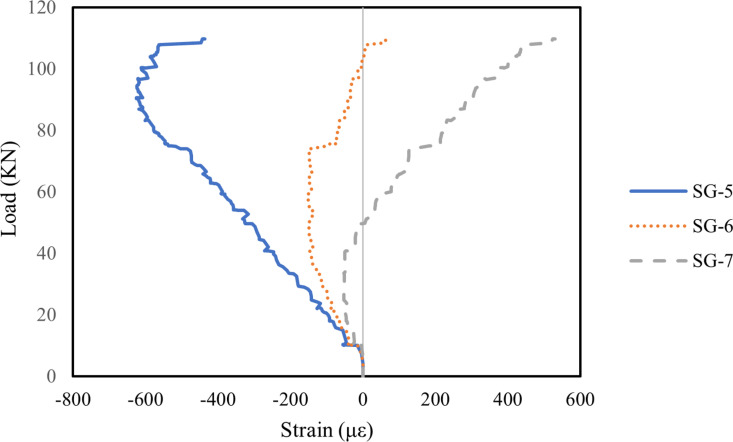
Strain on midspan of concrete in B2 beam.

**Fig 12:**
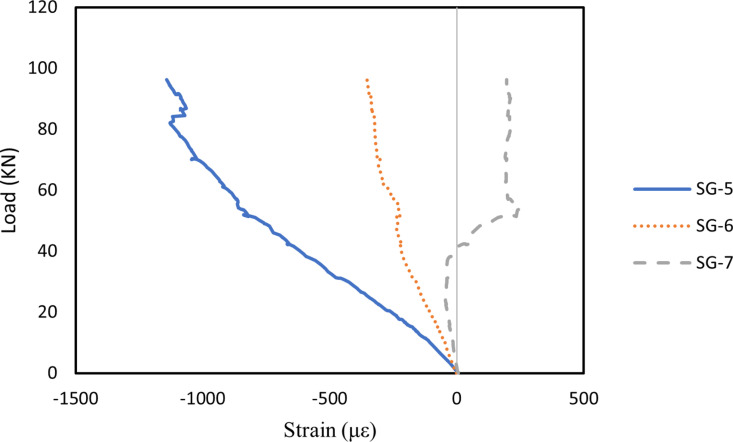
Strain on midspan of concrete in B3 beam.

In this study, strains in the compression zone of the RHA RC beams were also analysed, with Figs 10–12 illustrating the midspan concrete strains for all beams. The strain patterns in the compression zone were generally consistent across all beams. Strain gauge SG-5, positioned at the top of the beam, recorded the highest compressive strain due to the influence of the bending moment. Meanwhile, SG-6 and SG-7 indicated that compressive strain decreased as the load increased, eventually transitioning to tensile strain. Notably, the addition of well-dispersed MWCNTs in Beam B2 reduced strain in the RHA RC beam, whereas an excess of MWCNTs in Beam B3 resulted in increased strain. This effect is primarily attributed to the ability of well-dispersed MWCNTs to enhance the stiffness of RHA RC beams. Similar results were reported by Shi et al. [92], where RC beams incorporating basalt fibre-reinforced polymer and epoxy resin modified with MWCNTs exhibited reduced midspan strain compared to those reinforced with pure epoxy resin and basalt fibre-reinforced polymer. This effect is due to the improved stiffness of MWCNTs-modified epoxy resin, which reduces deformation and strain.

#### 3.5.1. Strain on side of concrete.

**Fig 13:**
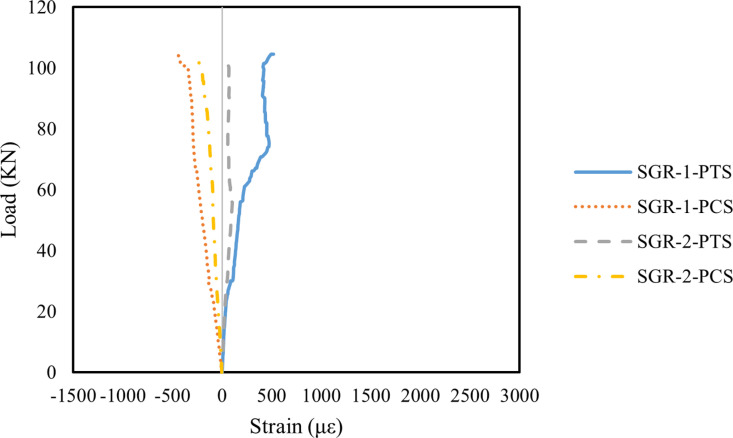
Principal strain on the side of B1 beam.

**Fig 14:**
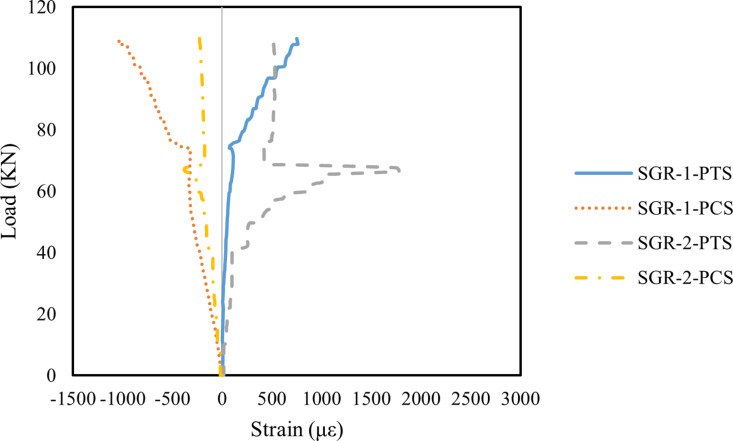
Principal strain on the side of B2 beam.

**Fig 15:**
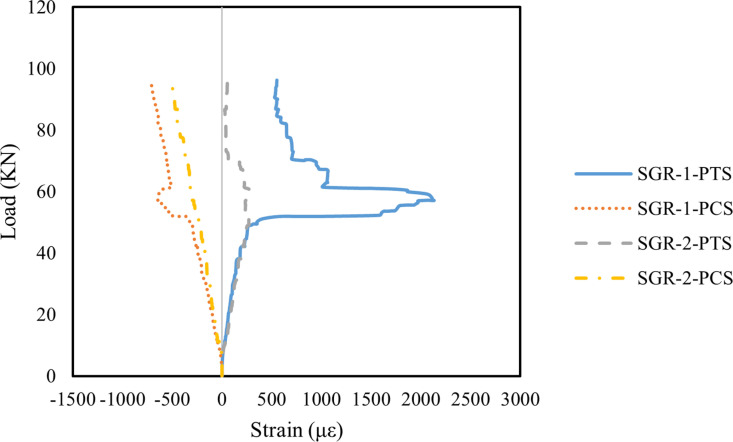
Principal strain on the side of B3 beam.


Figs 13–15 illustrate the principal strain distribution on the side of the beams. Since the reinforcement layout and measurement point arrangements on the left and right sides of the test beams are identical, the analysis below focuses on the measurement points on the left side. The principal strain values and directions were calculated using the following formula:

$$\varepsilon =\frac{\varepsilon_{x}+\varepsilon_{y}}{2}\pm \sqrt{{\left(\frac{\varepsilon_{x}-\varepsilon_{y}}{2}\right)}^{2}+{\left(\frac{\varepsilon_{x}+\varepsilon_{y}}{2}-\varepsilon_{45^{0}}\right)}^{2}},$$

$$\tan(2\theta )=\frac{\varepsilon_{x}+\varepsilon_{y}-2\varepsilon_{45^{0}}}{\varepsilon_{y}-\varepsilon_{x}},$$

where $\varepsilon_{x}$ is the strain in the horizontal direction, $\varepsilon_{y}$ is the strain in the vertical direction, $\varepsilon_{45^{0}}$ represents the strain at an angle of 45° to the horizontal direction, and θ is the angle between the principal strain and the horizontal direction. A positive calculation result indicates principal tensile strain (PTS), while a negative value denotes principal compressive strain (PCS).

As shown in Fig 13, the principal strains at SGR-1 and SGR-2 in Beam B1 increased steadily with the applied load. When the load was below 30 kN, the strain values remained low, indicating an elastic working state. However, with continued loading, an inflection point appeared in the SGR-1 PTS curve, where the slope slightly decreased. At 60 kN, a distinct inflection point was observed at SGR-1, which occurred because a crack passed through the SGR-1 measurement point, causing a sudden change in the principal strain. The primary failure cracks in Beam B1 were concentrated in the right span of the beam, whereas the crack width on the left side was significantly smaller. The crack in the left span only extended to the edge of the SGR-2 measurement point, resulting in a considerably lower principal strain at SGR-2 compared to SGR-1.

As shown in Fig 14, Beam B2 exhibited an extended elastic working stage compared to Beam B1. In particular, the SGR-2 measurement point remained in the elastic working stage when the applied load was below 73 kN. Additionally, during the elastic phase, the principal strain at the SGR measurement points in Beam B2 was lower than in Beam B1, indicating that the addition of MWCNTs improved the initial stiffness of Beam B2. Since the primary failure crack in Beam B2 was located in the left span, a noticeable inflection point occurred in the principal strain curve when the crack passed through the SGR measurement point. Specifically, at 60 kN, the principal strain at the SGR-2 measurement point exhibited a distinct turning point. The main inflection point for the SGR-1 measurement point appeared at 73 kN, after which the strain developed rapidly. These results suggest that the incorporation of MWCNTs delayed the onset of the principal strain inflection points, primarily due to the bridging effect of MWCNTs, which effectively slowed crack propagation [93].

As shown in Fig 15, the principal strain inflection point at the SGR measurement points of Beam B3 appeared earlier than in Beam B2. At 50 kN, the principal strain at the SGR-1 measurement point distorted and increased rapidly, indicating that a crack had passed through this location. Additionally, during the elastic working stage, the principal strain at the SGR measurement points in Beam B3 was higher than in Beam B2. This suggests that the initial stiffness and shear capacity of Beam B3 were significantly reduced, primarily due to excessive MWCNTs incorporation. The high MWCNTs concentration led to particle agglomeration, further compromising the mechanical properties of the concrete [69,94].

### 3.6. Comparison of experimental and predicted shear strengths

In this study, the most widely used shear capacity prediction models were selected for comparison with the ultimate shear capacity obtained from the experiments. These models include the ACI 318 model [95], Eurocode 2–05 model [96], and Canadian standard model [97], as well as the Bazant and Kim model [98], Zsutty model [99], and Collins and Kuchma model [100]. The equations used for each prediction model are presented in Table 6.

**Table 6:** Equations of prediction models.

Model	Equation
ACI 318 model	$V_{c}=0.17\sqrt{{f^{\prime }}_{c}}bd$ Where $V_{c}$ is the shear strength, ${f^{\prime }}_{c}$is the compressive strength, b is effective width, and d is the effective depth.
Eurocode 2–05 model	$V_{c}=[0.18K{\left(100\rho_{1}{f^{\prime }}_{c}\right)}^{\frac{1}{3}}]bd$ Where $\rho_{1}$ is the longitudinal reinforcement ratio. $K=1+\sqrt{\frac{200}{d}}$
Canadian standard model	$V_{c}=0.2\sqrt{{f^{\prime }}_{c}}bd$
Bazant and Kim model	$V_{c}=[0.54\sqrt[{3}]{\rho_{1}}\;(\sqrt{{f^{\prime }}_{c}}+249\sqrt{\frac{\rho_{1}}{({\frac{a}{d})}^{3}}}\left(\frac{1+\sqrt{\frac{5.08}{d_{a}}}}{\sqrt{1+\frac{d}{25d_{a}}}}\right)]bd$ Where $d_{a}$ is maximum size of coarse aggregate.
Zsutty model	$V_{c}=2.11{\left(\frac{{f^{\prime }}_{c}\rho_{1}d}{a}\right)}^{1/3}bd$
Collins and Kuchma model	$V_{c}=\frac{245}{1257+35(0.9d)/(d_{a}+16)}\sqrt{{f^{\prime }}_{c}}\;bd$

**Fig 16:**
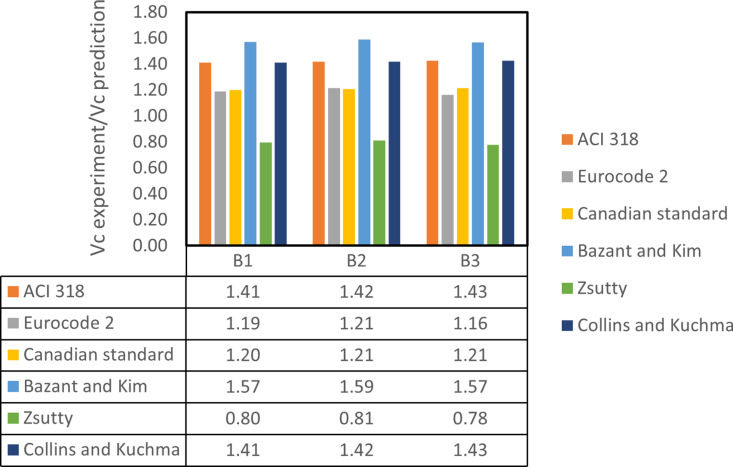
Vc experiment/prediction ratio of all beams.

Table 7 provides a comparison of the experimental and predicted shear capacities for each beam, while Fig 16 illustrates the experiment-to-prediction ratios across all beams. As shown in Fig 16, nearly all prediction models—except the Zsutty model—overestimated the ultimate shear capacity. While the predicted values for all models increased with MWCNTs content, an excessive concentration of MWCNTs resulted in lower predicted values. The average shear strength experiment-to-prediction ratios ($V_{c}$ experiment/prediction) for the Bazant and Kim, ACI 318, and Collins and Kuchma models were 1.58, 1.42, and 1.42, respectively. In contrast, the predictions from the Canadian standard and Eurocode 2–05 models were more closely aligned with experimental results, with average ratios of 1.21 and 1.19, respectively. The Zsutty model was the only one to underestimate the ultimate shear capacity; however, its predictions were closer to the experimental values than those from the ACI 318, Collins and Kuchma, and Bazant and Kim models, yielding an average experiment-to-prediction ratio of 0.8. Overall, for predicting the ultimate shear capacity of RHA concrete incorporating MWCNTs, the Zsutty, Eurocode 2, and Canadian standard models proved to be the most reliable, with an approximate prediction error of 20%.

**Table 7:** Results of experimental V_c_ and predicted V_c_.

Beam	Exp. $V_{c}$ (KN)	Predicted $V_{c}$ (KN)					
		ACI 318	Eurocode 2	Canadian standard	Bazant and Kim	Zsutty	Collins and Kuchma
B1	52.5	37.17	44.09	43.72	33.39	65.84	37.20
B2	55	38.74	45.33	45.58	34.63	67.69	38.78
B3	48	33.63	41.24	39.56	30.61	61.59	33.66

## 4. Conclusion

This study investigated the shear behaviour of RHA RC beams incorporating varying concentrations of MWCNTs (0–0.2%) by evaluating crack propagation, failure modes, load-deflection behaviour, and strain distribution in both longitudinal reinforcement and concrete. Additionally, the experimental results were compared with existing shear design models. Based on a comparative analysis of RHA RC beams with MWCNTs (Beams B2 and B3) and without MWCNTs (Beam B1), the following conclusions were drawn:

i The addition of well-dispersed MWCNTs enhanced the shear resistance of RHA RC beams. Crack propagation was effectively delayed, and crack widths were significantly reduced.
ii With the incorporation of 0.1% MWCNTs, the first crack load, initial stiffness, and ultimate shear capacity increased by 34.78%, 38.4%, and 4.76%, respectively, compared to Beam B1.
iii Midspan deflection analysis demonstrated that the inclusion of MWCNTs significantly improved beam ductility. The midspan deflection at the first crack load and ultimate load in Beam B2 increased by 9.47% and 7.69%, respectively, compared to the control specimen. Additionally, Beam B2 exhibited a 23.19% improvement in ductility relative to Beam B1.
iv The addition of 0.1% MWCNTs effectively delayed the rapid development of strain in the longitudinal reinforcement, significantly reduced strain in the compression zone of the RHA concrete beam, and postponed the occurrence of the principal strain inflection point on the beam’s side.
v Excessive incorporation of MWCNTs (0.2%) negatively impacted shear resistance, reducing the ultimate shear capacity by 8.57%. Although the deflection of Beam B3 was similar to that of Beam B2, the ultimate load required to reach this deflection was approximately 87.3% of that of Beam B2. Moreover, the strain in the compression zone of Beam B3 developed more rapidly than in Beam B2, and the principal strain inflection point appeared earlier.
vi Among the shear strength prediction models, the Zsutty model was the only one that did not yield conservative estimates. The Eurocode 2 and Canadian standard models exhibited the smallest prediction errors (approximately 20%) and were the most suitable for predicting the shear strength of RHA RC beams.

This study demonstrates that incorporating MWCNTs can effectively enhance the shear resistance of RHA RC beams, addressing the existing research gap regarding the shear behaviour of RHA-based sustainable concrete beams. In order to prevent the shear failure of RHA RC beams, the optimal dosage of MWCNTs is recommended to be 0.1%. The well-dispersed MWCNTs contribute to preventing the brittle failure of RHA RC beams when subjected to shear pressure. Exceeding 0.2% MWCNTs is inadvisable, as excessive inclusion can result in the reagglomeration of MWCNTs and diminish the mechanical characteristics of RHA RC beams. The findings further highlight the potential of MWCNTs in developing high-performance RHA sustainable concrete structural components, providing an experimental foundation for the broader application of RHA concrete in the construction industry.

This study has a few limitations. First, the effect of RHA produced under different combustion conditions on the shear performance of RHA concrete beams was not considered. Second, the influence of MWCNTs with varying lengths and preparation techniques on the shear performance of RHA concrete beams was not examined.

For future research, the following studies are recommended: First, investigating the effect of RHA content on the performance of concrete materials and structural members. Second. evaluating the performance of structural members containing RHA and MWCNTs under extreme conditions, such as high temperatures. Third., evaluating the impact of different types of MWCNTs on the performance of structural members incorporating RHA.

## Supporting information

S1 Table Diagonal crack width and nominal shear stress. (XLSX)

S2 Table Load and deflection results of all beams. (XLSX)

S3 Table Steel bars strain results of B1 beam. (XLSX)

S4 Table Steel bars strain results of B2 beam. (XLSX)

S5 Table Steel bars strain results of B3 beam. (XLSX)

S6 Table Strain results on midspan of concrete in B1 beam. (XLSX)

S7 Table Strain results on midspan of concrete in B2 beam. (XLSX)

S8 Table Strain results on midspan of concrete in B3 beam. (XLSX)

S9 Table Principal strain results on the side of B1 beam. (XLSX)

S10 Table Principal strain results on the side of B2 beam. (XLSX)

S11 Table Principal strain results on the side of B3 beam. (XLSX)
